# The Active Segmentation Platform for Microscopic Image Classification and Segmentation

**DOI:** 10.3390/brainsci11121645

**Published:** 2021-12-14

**Authors:** Sumit K. Vohra, Dimiter Prodanov

**Affiliations:** 1 Visual and Data-Centric Computing, Zuse Institute Berlin (ZIB), Takustrasse 7, 14195 Berlin, Germany; vohra@zib.de; 2 Neuroscience Research Flanders (NERF), Interuniversity Microelectronics Centre (IMEC), Kapeldreef 75, 3001 Leuven, Belgium; 3 Information Technologies for Sensor Data Processing (ITSDP) Department, Institute of Information and Communication Technologies (IICT), Bulgarian Academy of Sciences, Acad. G. Bonchev St., Block 25A, 1113 Sofia, Bulgaria

**Keywords:** interactive segmentation, random forest, support vector machines, features exploration, scale space, differential invariants, legendre moments, zernike moments

## Abstract

Image segmentation still represents an active area of research since no universal solution can be identified. Traditional image segmentation algorithms are problem-specific and limited in scope. On the other hand, machine learning offers an alternative paradigm where predefined features are combined into different classifiers, providing pixel-level classification and segmentation. However, machine learning only can not address the question as to which features are appropriate for a certain classification problem. The article presents an automated image segmentation and classification platform, called Active Segmentation, which is based on ImageJ. The platform integrates expert domain knowledge, providing partial ground truth, with geometrical feature extraction based on multi-scale signal processing combined with machine learning. The approach in image segmentation is exemplified on the ISBI 2012 image segmentation challenge data set. As a second application we demonstrate whole image classification functionality based on the same principles. The approach is exemplified using the HeLa and HEp-2 data sets. Obtained results indicate that feature space enrichment properly balanced with feature selection functionality can achieve performance comparable to deep learning approaches. In summary, differential geometry can substantially improve the outcome of machine learning since it can enrich the underlying feature space with new geometrical invariant objects.

## 1. Introduction

Segmentation of cells and sub-cellular structures (e.g., organelles) is a frequent intermediate step in cell biology and pathology [1,2]. For example, in digital pathology, detection and segmentation of nuclei is one of the core operations in image analysis [3]. Similar is the situation in plant microscopy [4]. Image segmentation can be broadly defined as isolating objects of interest from an image by either a manual or automatic algorithmic labeling. Automated approaches for cell segmentation have a long history, without ever reaching a universal solution [1]. This is far from a surprise, because the underlying physical modalities are very diverse. Sample preparation can be also quite different, depending on the particular experimental question—i.e., light microscopy using colored histological stains, which is still the standard in pathology; vs. fluorescent microscopy using various fluorescent stains or genetic fluorescent markers, such as the green fluorescent protein GFP; vs. transmission electron microscopy (TEM) using heavy metal contrasts (for example osmium) for sub cellular structures; vs. phase contrast microscopy for live cell imaging.

The majority of traditional cell segmentation methods are based on only a few basic approaches. Following in part Mejiering [1] these can be classified into:

- histogram based
- edge-based
- morphological operations
- region-based, such as watersheds
- deformable model fitting

Algorithms tailored for one imaging modality are very likely to fail in other modalities. For example, intensity thresholding completely fails if there is a large intensity variation of the image background, for example in a dense tissue sample. Various pre-processing steps, such as morphological top-hats of high-pass filtering can be implemented, but they are only suitable for images, which are sufficiently similar to the test batches for which the pre-processing is optimized [5].

On the other hand, the manual segmentation is labor intensive and prone to intersession and inter-observer variations. The segmentation of microscopic images is a non-trivial problem. While in the past such segmentation was performed by experts, the demands and intensity of the present experimental approaches makes such an approach non-viable. While simple protocols, such as cell counting (e.g., in a live/dead assay), can be addressed adequately by approximate segmentation, this is far from a complete morphological characterization, where the shape and orientation are to be quantified. Cells frequently have complex shapes, examples here include astrocytes, microglia, dendritic cells, etc. On the second place, in some cases, structures within some cell types can be of interest—i.e., mitochondria, synaptic vesicles, nuclei etc. As an example of the traditional approach can be identified NucleusJ [6]. It has a specific application targeting nuclear segmentation, morphometry and statistical analysis of the nucleus.

The question then arises how can the expert knowledge be harnessed by computer algorithms in the best way in order to develop versatile segmentation solutions able to address a wide range of segmentation problems. Ideally, such an approach would be

(1) deterministic, in order to ensure reproducibility of the results;
(2) providing an uncertainty measure of the segmentation outcome;
(3) able to provide some insight into how the morphology is objectively described
(4) able to learn new information whenever such is presented

At present, there are several available platforms covering parts of the above requirements—Trainable Weka Segmentation (TWS) [7] (part of Fiji), Ilastik [8], Icy [9] and CellProfiler [10]. An interesting development is the QuPath platform [11]. It targets digital pathology as a main application. The platform was specifically designed to analyze and explore whole slide imaging data consisting of image tiles. This is, of course, a different path compared to ImageJ and Ilastik, where the planar image is a compact object.

Texture features have been used to classify images since 1970s [12]. Recent applications include [3,13]. In a similar way, image classification can be classified into 3 main categories [14]:

- numerical feature engineering method (NFE);
- Artificial Neural Networks (ANNs);
- Transport based morphometry (TBM);

The use of NFE is the traditional way to classify images. Based on expert knowledge or automatic algorithms, several morphological and polynomial features are extracted an image. Once the features are computed, classification algorithms, such as Support Vector Machines (SVM), or random forests, are then applied to the feature vectors. The main advantage of using NFE over neural networks are that the former algorithms are fast and do not need supervision. On the other hand, ANNs bypass the step of feature extraction and use several fully connected convolution layers to learn features from the source images.

There are numerous open-source programs for biomedical image processing. This can be attributed in part to the fact that in the past every imaging facility developed its own analysis tools. The situation changed gradually after 2005 with the gaining of popularity of ImageJ and its offshoots Fiji and ImageJ2 [15], so at present the ImageJ tool ecosystem is a *de facto* standard for biological applications [16,17]. What made ImageJ ecosystem unique is the fact that the original ImageJ design was minimalistic, focusing only on the essential features, which could nevertheless be extended in almost any direction by contributed plugins that seamlessly integrate into the main user interface. So that without emphasis on programming paradigms and particular frameworks the entry point for new users and developers remained low. Further development of the ImageJ family included Fiji [18], where one of the authors (D.P.) was an early contributor, as well as CellProfiler [10], Icy [9], and ImageJ2 [15]. All these platforms have varying entry thresholds for new users and developers. Therefore, to truly make an impact, a new solution should be at least interoperable with some of these platforms or preferably be implemented in one of them.

The aim of the present article is to demonstrate a useful addition to the ImageJ tool ecosystem. The Active Segmentation for ImageJ (AS/IJ) has been developed since 2016 under the auspices of the Google Summer of Code Program as an activity of the Belgian Neuroinformatics Node, affiliated to the International Neuroinformatics Coordination Facility (INCF). AS/IJ is not a mere incremental improvement of TWS, which served as an inspiration and starting point of the project. The platform architecture exploits ImageJ’s modularity of design and even goes one step further: filters, compatible with the platform, are themselves plugins of AS/IJ. They could even be independent ImageJ plugins, although this is not necessary. AS/IJ supports rich and extensible metadata. The metadata structure ensures reproducibility of the classification and segmentation results across sessions. This design choice is made specifically to ensure support of the Findability, Accessibility, Interoperability, and Reusability (FAIR) data management principles. In particular, we implement the R-part of the principle in the way that all filtering settings are stored in the project file in JavaScript Object Notation (JSON) format. Furthermore, the file format is transparent for both human eyes and algorithms as JSON is a subset of the standard web language JavaScript.

The present paper applies the AS/IJ platform on two related problems: automatic classification and segmentation of microscopic images. We have employed generic approaches applicable to a wide variety of microscopic images by leveraging the power of differential geometry combined with scale space theory. The paper is organized as follows. Section 2 presents the minimal necessary background on differential geometry, scale space theory, image moments, and machine learning. Section 3 describes the user interface and data format. Section 4 describes the used data sets and the setting of the comparison tasks. Section 5 focuses on the comparison with Ilastik [8]. Finally, Section 6 discusses our design choices and the obtained results.

## 2. Theory

Typically, the planar images are represented as surfaces in the three-dimensional Euclidean space $E^{3}$, where the elevation represents the signal intensity. While good for visualization purposes, such representation can be overly restrictive. In particular, one needs not presuppose the Euclidean character of the embedding space. Mathematically, it is more fruitful to treat the image as embedded in an abstract n+2-dimensional vector space, which is a product of the Euclidean image plane with the vector space, provided by the point-wise image features: $I^{\prime }=E^{2}\times V_{n}$. In order to fully harness the powerful theory of differential geometry, certain smoothness conditions must be imposed on the data.

### 2.1. Geometrical Image Features

The fact that digital images are sampled on a discrete grid may represent some difficulty, as differentiation in the literal sense is not applicable to discrete signals. Notably, naive computations using finite differences are numerically unstable and amplify the high frequency noise. This difficulty can be overcome by applying the distribution theory [19], using the definition of distributional gradient (see also Appendix A). Therefore, even for discrete images, by extension, one can define a weak differentiation operations in distributional sense in terms of convolution with the gradient of a smooth kernel function as:

(1) $$\nabla_{G}I:=-I*\nabla G$$

where the symbol ∇ represents the of the gradient given by its principal components ∇=(∂/∂x,∂/∂y). This approach can be applied also to subsequent differentiation operations, in this way enriching the feature space.

Consider a smooth scalar planar image I(x), where x is the radius-vector. The image intensity at a certain point in the direction x+r can be interpolated from its local neighborhood up to second order as

$$I\left(x+r\right)=I\left(x\right)+r\cdot \nabla_{G}I+\frac{1}{2}r^{T}\cdot H_{G}\left(I\right)\cdot r+O\left(r\cdot r\right)$$

where the dot represents matrix multiplication. The symmetric Hessian tensor H is represented by the matrix of second order partial derivatives. By analogy, the Hessian of Gaussian (HoG) tensor $H_{G}$ is represented by the matrix of the convolutions of the second partial derivatives of the kernel *G*, thus representing a weak definition of the Hessian (see also Appendix A). This picture is a part of the image *jet-space*, which is a higher dimensional differential vector space, as a natural basis for encoding the geometry of an image point local neighborhood [19,20].

### 2.2. Differential Invariants

There are several types of geometric features, which are useful for segmentation applications. Typical interesting image features are blobs, filaments and corners. Notably, object boundaries can be represented in terms of edges, which can be approximated by steps in image intensity. All these features can be computed from the local differential structure of the image and notably from the differential invariants. The theory will be exemplified with the Gaussian derivatives, which in view of the duality property of Equation (1), can be used to compute the image derivatives. The first four differential invariants are given in Table 1.

The eigenvalues of the Hessian tensor are solutions of the characteristic equation det(H−λI)=0, where I is the identity matrix. This is a square equation with two real roots $\lambda_{1,2}$, such that $\lambda_{1}+\lambda_{2}=\Delta_{G}$ and $\lambda_{1}\lambda_{2}=det\;H_{G}$. In addition, by the symmetry of the Hessian, the eigenvectors form 2 mutually orthogonal vector fields.

If both eigenvalues are negative, this is an indication for a bright blob-like feature around the point of reference. In a similar way, if both eigenvalues are positive, there is a dark blob-like feature around the point of reference. If the eigenvalues have opposite signs this is an indication of a saddle point at this point. Therefore, the zero-crossing of the Laplacian operator can be used to delimit regions, encompassing blobs. The zero-crossings form the so-called “zero space”. This can be applied also to the normal component of the ALoG and can be used to segment blobs [21].

The number of differential invariants increases with the increase of the image dimensions, but the theory can be extended along similar lines.

### 2.3. Curvature Invariants

Second-order properties of smooth surfaces are described well by two invariants of interest—mean curvature and Gaussian curvature, while of lines—by one invariant—the line curvature. Geometrically, the mean curvature is given by the divergence of the unit normal vector. While the Gaussian curvature is the limit ratio of the solid angle subtended by the normal projection of a small surface patch divided by the area of the patch. The formulas are presented in Table 2. The curvature invariants are implemented as two filters—Curvature 2D and Curvature 3D (see Table 3).

### 2.4. Structure Tensor

The structure tensor (ST) is an abstract extension of the gradient. The tensor encodes the predominant directions of the gradient in a specified neighborhood of a point, and the degree to which those directions are coherent as a function of scale. Suppose that we have a scale-space representation of the gradient $\nabla_{G}$. Then the structure tensor is the smoothed tensor product of the smoothed gradient vector [22]:

(2) $$S_{r}\left(I\right):=G_{r}*\left\{\nabla_{G}I\cdot \nabla_{G}I^{T}\right\}$$

From this expression it is apparent that the operator introduces smoothing on two scales. However, because of the operator’s quadratic character the scales do not compose. $S_{r}\left(I\right)$ can be represented by a 2 × 2 matrix. Besides the information on orientation and magnitude of structures, which is already present in the gradient, the structure tensor contains some additional information. This additional information is produced by the second smoothing step and measures the homogeneity of gradient field orientations within the neighborhood of a pixel. The information contained in the tensor is encoded by its eigenvalues and eigenvectors. Suppose that there is no second smoothing step. Then, the maximal eigenvalue encodes the eigenvector in the direction of the gradient, while by symmetry the second eigenvector is orthogonal and is in the direction of the isophote. So that the encoded information is equivalent to the gradient. Moreover, if the second eigenvalue is 0 it means that the image has constant intensity along the direction of the second eigenvector. The second smoothing step induces a weighted averaging in a larger window. A useful measure of the homogeneity of the image is the coherence

(3) $$c={\left(\frac{\lambda_{1}-\lambda_{2}}{\lambda_{1}+\lambda_{2}}\right)}^{2}$$

This quantity is 1 when the gradient field is totally aligned, and 0 when it has no preferred direction. The structure tensor is implemented as the filter Gaussian Structure (see Table 3).

### 2.5. Scale Space Theory

In the discrete domain, smoothing leads to loss of resolution and, therefore, of some information. However, the information loss can be limited if one uses multiple smoothing scales. A systematic way to treat such information loss is offered by the scale space theory [23,24]. The axiomatic linear scale space theory was formulated in series of works by Witkin and Koenderink [25,26]. In its original version, the theory depends on several properties of the Gaussian filters as solutions of the diffusion equation in the scale-space generated by the image. That is, the generic smoothing kernel *G* is identified with a radially-symmetric Gaussian kernel of scale $s=\sigma^{2}\in R$

(4) $$G\left(r\right)=\frac{e^{-r^{2}/2s}}{2\pi s}$$

The Gaussian kernels provide several advantages: (i) they are rotationally invariant (ii) they do not produce artificial extrema in the resulting image (iii) successive convolutions with different kernels can be combined. Mathematically, this imposes a very useful semi-group structure, equivalent to the heat/diffusion equation. In this sense, the image structures diffuse or “melt-down”, so that the rate of this diffusion indicates the “robustness” of the structure. In its typical presentation, the scale space theory applies only smoothing steps. Later, the theory was extended to include also differentiation and thus account for the differential structure of the images [27].

Linear diffusion scale-spaces are well-posed and have a solid axiomatic foundation. On the other hand, for some applications, they have the undesirable property that they do not permit contrast enhancement and that they may blur and delocalize structures. Non-linear scale spaces try to overcome some of these limitations. Such scale spaces arise in non-linear partial-differential equation framework. The formal properties of some types of scale spaces have been established by Alvarez et al. [28] and encompass anisotropic diffusion, among other approaches.

In particular, the Laplacian operator can be decomposed into two mutually orthogonal components: one along the direction of the gradient and another one along the direction of the isophote ([29], Chapter 1; see Appendix B). In a similar way, the Laplacian operator can be iterated to give rise to the Power-of-Laplacian (PoL) operator [21]:

(5) $$\Delta_{G}^{n}I=\left(\underset{n\;\mathrm{times}}{\underset{︸}{\Delta_{G}\ldots }}\right)I$$

This operator enhances high-frequency features of an images given the scale cut-off. This can be seen easily from the frequency response of the LoG filter $F_{k}\Delta_{G}=-k^{2}s^{2}$ where *k* is the wave vector and $F_{k}$ is the symbol of Fourier transform. The PoL operator is immediately generalizable from its Fourier space representation to the positive real domain of the exponent. This can be achieved in terms of the α-scale space theory [30], which offers interesting possibilities of a broader class of smoothing filters, which are derivable from the Riesz Laplacian operator. The PoL operators are implemented by 2 filters—BoG and LoGN for speed reasons (see Table 3).

### 2.6. Image Moments

In the continuous approximation, the moments of a planar function are defined by the integral

$$M_{m,n}:=\underset{I}{\;\int \int \;}P_{m,n}\left(x,y\right)f\left(x,y\right)dxdy$$

where $P_{m,n}\left(x,y\right)$ is polynomial, parameterized by the integers *m* and *n*. For example, the raw image moments are given by the homogeneous form $P_{m,n}\left(x,y\right)=x^{m}y^{n}$. The moments, can be referred to the center of the image frame or to the center of mass of the image $\left(x_{c},y_{c}\right)$, in which case, $P_{m,n}\left(x,y\right)={\left(x-x_{c}\right)}^{m}{\left(y-y_{c}\right)}^{n}$. The two main problems with such a choice is that the moments contain redundant information because the homogeneous polynomials are not orthogonal; also the computation loses numerical precision due to cancellation of large terms. Mathematically, a better choice of polynomials is a polynomial from an orthogonal family. Such polynomials enjoy an expansion property, that is

$$f\left(x,y\right)=\sum_{m=0}^{\infty }\sum_{n=0}^{\infty }M_{m,n}P_{m,n}\left(x,y\right)$$

Useful examples of such orthogonal families are the Legendre and Zernike polynomials.

The Legendre polynomials form an orthogonal set on the interval [−1,1] The Legendre polynomials enjoy a two term recurrence relation

(6) $$\left(n+1\right)L_{n+1}\left(x\right)=\left(2n+1\right)xL_{n}\left(x\right)-nL_{n-1}\left(x\right),\;L_{0}\left(x\right)=1,\;L_{1}\left(x\right)=x$$

that was used for their computation in the present paper. The advantage of the recursion relation is that all Legendre moments up to a given order can be computed simultaneously.

The Zernike polynomials are normalized on the unit disk in the complex plane. The radial Zernike polynomials can be defined for n−m even as:

(7) $$R_{n}^{m}\left(r\right):=\sum_{l=0}^{(n-m)/2}\frac{{\left(-\right)}^{l}\left(n-l\right)!}{l!((n+m)/2-l)!((n-m)/2-l)!}r^{n-2l}$$

and 0 otherwise. The present paper implemented a recursive computation method given by the formula [31]

(8) $$R_{n}^{m}\left(r\right)=r\left(R_{n-1}^{\left|m-1\right|}\left(r\right)+R_{n-1}^{m+1}\left(r\right)\right)-R_{n-2}^{m}\left(r\right),\;R_{0}^{0}=1$$

The orthogonal Zernike polynomials then are defined as

(9) $$V_{mn}\left(r,\theta \right):=R_{n}^{m}\left(r\right)e^{-im\theta },$$

where *i* is the imaginary unit. The normalization of the polynomials for grayscale images is not an issue because they have a fixed dynamic range, so an image can be always normalized to unit range prior to computation of an image moment.

### 2.7. Random Forest Classifier

The random forests are an ensemble learning method for classification, which consists of a large number of individual decision trees [32]. For classification tasks, the output of the random forest is the class selected by most trees by majority voting. It is one of the most used algorithms, because of its simplicity and diversity (it can be used for both classification and regression tasks). The random forest selects features at random and it searches for the best feature among this random sample of features. The random forest classifier is preferred over other nonlinear classifiers due to a fewer number of hyper parameters—i.e., number of trees for segmentation and number of features for splitting. By default, for a segmentation problem with *k* features, 100 trees are used to learn the model and $\sqrt{k}$ are used for feature splitting.

### 2.8. Support Vector Machines Classifier

The Support Vector Machines (SVM) is a supervised machine learning algorithm, which is a generalization of the one-dimensional perceptron [33]. The algorithm is used to classify incoming data by calculating a hyperplane, maximizing the distance from the nearest data point on each side. If such a hyperplane exists, it is denoted as the maximum-margin hyperplane. Several implementations of SVMs are available i.e., Sequential Minimal Optimization (SMO) [34], LibLinear [35] and LibSVM [36]. We have used SMO [34] because it is fast and divides the large quadratic optimization problem into sub problems and uses heuristic to solve each sub-problem. In order to select an SVM implementation, we have compared all the three implementations on HeLa’s data set. The obtained results were similar (not shown).

## 3. User Interaction and Architecture

### 3.1. Architecture

The Active Segmentation/ImageJ platform is implemented as a plugin for the public domain image analysis platform *ImageJ*. AS/IJ is fully integrated with ImageJ. As can be seen from the theoretical overview, an image filter transforms an image point to another point in a derivative image, therefore in mathematical terms it is a 1:1 transformation (injective transform). On the other hand, an image moment transforms an entire image region to a point, therefore it is N:1 (surjective transform). This distinction imposes some differences in the programming architecture. The filters implement the *IFilter* interface, while the surjective transforms implement the *IMoment* interface. The filtering functionality of the platform is extendable via plugins, which are loaded automatically upon starting of the platform from the plugin path. The built-in filters are listed in Table 3. Every filter is a fully functional ImageJ plugin, which can be also used in a standalone mode.

The signal processing approach demonstrated in the paper is based on the scale space theory [23], as a systematic way of treating image features based on differential invariants [37]. However, the software implementation is not limited by this approach and can handle any injective and surjective image transforms. The machine learning functionality is based on the Waikato Environment for Knowledge Analysis (Weka) library, version 3.9.

### 3.2. Operation of the Platform

**Figure 1:**
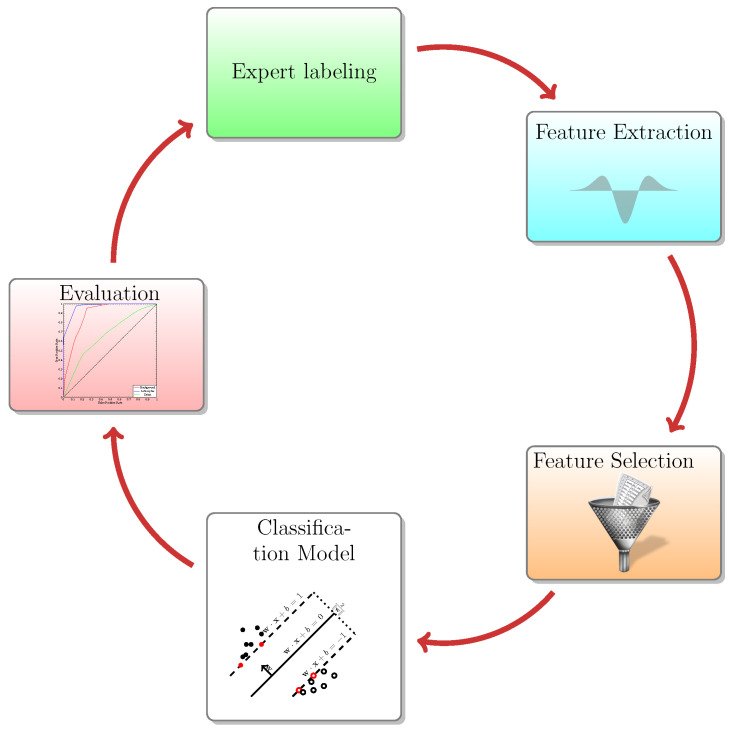
Learning cycle.

**Figure 2:**
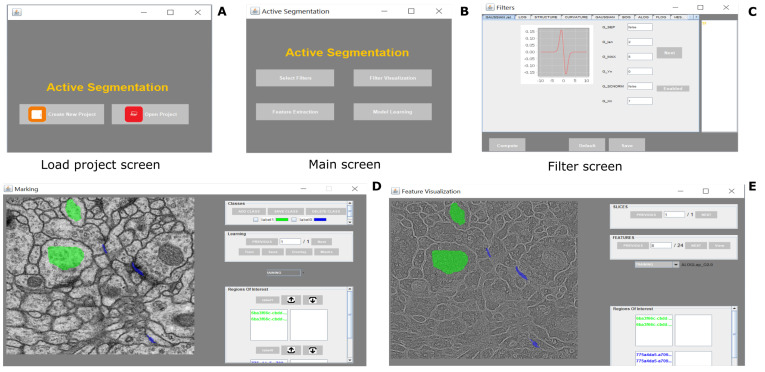
Segmentation and classification functionality of AS/IJ.

There are 2 user interaction modes: (1) for image segmentation or (2) for image classification. The users can initiate analysis project or load an existing one. If the users load an existing project they can (1) select suitable filters, (2) define classes and (3) present instances for learning (4) select machine learning functionality; and finally (5) train and visualize the achieved segmentation. This operation mode and the associated learning cycle is schematically presented in Figure 1. The user interface of the platform is displayed in Figure 2. As in TWS and Ilastik the user is expected to draw ROIs of various shapes (i.e., annotate) in order to present ground truth to the learning functionality (Figure 2D). The process can be repeated until a satisfactory result is presented. Different classes can be defined at will and associated with unique colors for better user experience. The outcome of the segmentation can be drawn on the selected feature view (Figure 2E). The orientation outputs comprise both the sine and cosine of the phase angle.

The image classification interaction mode is similar. The texture computing functionality is enabled by default for a classification project. The built in texture features are listed in Table 4. The functionality is also extendable by a different plugin interface. The feature-pluings are also loaded automatically from the plugin path.

Both segmentation and classification results can be fine-tuned by presenting additional ROIs or by refining and editing the existing ROIs. This allows a high degree of tunability of the outcome, which corresponds with practice. This feature has been borrowed from the TWS platform, which served as an inspiration for our work.

### 3.3. Metadata

The platform produces a large number of intermediate files, which are stored locally in a predetermined folder structure. This is described in the project metadata. The project metadata are stored in JSON format, which allows also for human readability and debugging. The file stored all filter settings, Regions of Interest (ROIs), and class labels allowing for reproducibility of the obtained results. A sample data set can be downloaded from https://zenodo.org/record/5771719 (see also Data Availability Statement).

**Figure 3:**
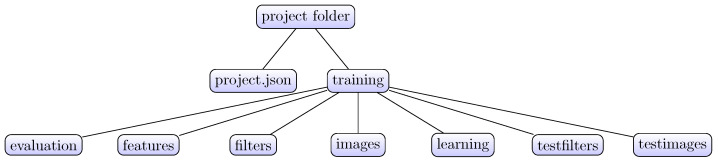
Project structure.

The AS/IJ project structure is exhibited in Figure 3. The “images” folder contains a copy of the training raw data (i.e., an image stack). The “features” folder contains all filter responses for the training data sets. The “learning” folder contains the Weka machine learning file in Attribute-Relation File Format (ARFF) format. The “tesfilters” folder contains all filter responses for the testing data sets. The “testimages” folder contains a copy of the testing raw data.

## 4. Materials and Methods

A brief remark on terminology is order. The term feature, which is generally accepted in classification and machine learning literature for the purposes of the present discourse is ambiguous. As noted above, an image filter is 1:1 (i.e., injective), therefore the generated features will be denoted as *point-features* or *pixel-features*, while features generated by a surjective operation (e.g., moment computation or similar) will be denoted as *regional features*, since they are derived from a ROI. For example, the standard ImageJ regional features are of two types: (i) geometrical, characterizing the primary ROI—area, bounding rectangle, centroid, perimeter, ellipse, Ferret’s diameter, shape descriptors (form factor, roundness, compactness, aspect ratio); and (ii) statistical, which are derived from the ROI histogram—mean, standard deviation, mode, min, max, center of mass, integrated density, median, skewness, kurtosis, and area fraction. In the subsequent discourse, the term geometrical will be interpreted only as referring to differential geometric properties.

### 4.1. Data Sets

#### 4.1.1. Synthetic Data Sets

Two synthetic data sets were used for demonstration and debugging purposes. Both data sets contain 5 images of size 256 × 256 pixels. One was used for the segmentation task and the other—for the classification task. The first data set consists of pairs of half ellipses, having the appearance of kidney beans, superimposed on a background with or without noise. The ellipses are placed in such a way as to only minimally overlap.

**Figure 4:**
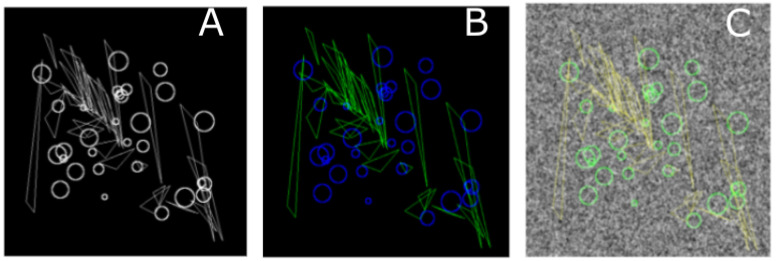
Circles/Triangles classification. (**A**) An image generated with variable size triangles and circles on black background; (**B**) Individual objects are classified as triangles (green) and circles (blue) on noiseless background; (**C**) Individual objects are classified as triangles (green) and circles (blue) on noisy background.

For the classification task data sets, circles of random sizes were placed randomly in the image plane, while the triangles were affinely transformed and also placed randomly. The motivation behind using these two classes of figures is that Zernike moments are more sensitive toward circles while Legendre moments are more sensitive toward lines. Figure 4A,C show representative images with and without background Gaussian noise.

#### 4.1.2. EM ISBI Challenge Data Set

Cardona et al. [38] published this data set for analysis of neuronal structures of the Drosophila ventral nerve cord. The raw data come from serial section Transmission Electron Microscopy (ssTEM). The data sets are annotated with the segmentation ground truth in the form of binary labels. The ground truth consists of 2 classes as shown in Figure 2 where the black color represents membranous structures and white represents other objects. This training data set contains 30 images of 512 × 512 pixels and measures 2 × 2 × 1.5 microns, with a resolution of 4 × 4 × 50 nm/voxel. The data set was included in the ISBI EM segmentation challenge (see sec. Data Availability). The images are representative of actual EM images and contain some noise and small image alignment errors. None of these problems led to any difficulties in the manual labeling of each element in the image stack by an expert human neuroanatomist. The aim of the challenge was to compare and rank the different competing methods based on their pixel and object classification accuracy. Besides the data set, the challenge defined evaluation metrics [39] based on membrane certainty and uncertainty in order to evaluate the performance of different classifiers.

#### 4.1.3. HeLa Data Set

**Figure 5:**
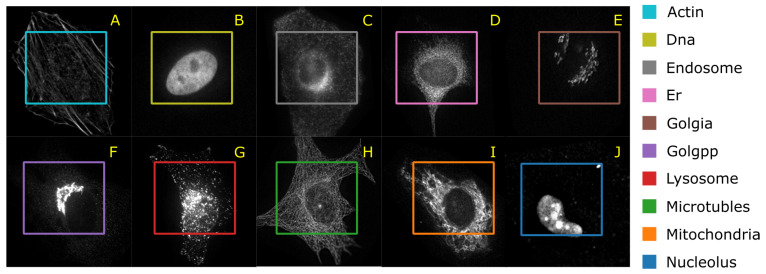
A representative image of each protein class and the average number of correctly classified instances in 10 cross validation (**A**) filamentous actin labeled with rhodamine-phalloidin (Actin); (**B**) DNA labeled with DAPI (DNA); (**C**) endoplasmic reticulum protein (ER); (**D**) transferrin receptor (endosome); (**E**) Golgi protein giantin (Golgia); (**F**) Golgi protein GPP130 (Golgpp); (**G**) lysosomal protein LAMP2 (lysosomes); (**H**) microtubules; (**I**) mitochondrial protein (mitochondria); (**J**) nucleolar protein nucleolin (nucleolus).

Murphy [40] and Boland et al. [41] have published the HeLa data set and investigated the role of expressed protein at subcellular level. As explained in the original publications, the data set contains 10 different classes of proteins—endoplasmic reticulum protein (ER); the Golgi proteins giantin and GPP130; the lysosomal protein LAMP2; nucleolar protein nucleolin; a mitochondrial outer membrane protein; the cytoskeletal proteins actin and tubulin; and the endosomal transferrin receptor. Figure 5 shows representative examples of each protein structure. This data set consists of 862 images of size 382 × 382 pixels, annotated with ground truth labels. These images were originally acquired in TCL format and have been converted to PNG image format. Each pixel is stored in 16 bit precision. The original images in the data set have been prepared as follows: (1) The original image stacks have been numerically deconvolved using the nearest neighbor algorithm. (2) They have been cropped so that there is one cell per image, and (3) all pixels outside this crop region have been set to 0 [40]. The data set is imbalanced with a smaller number of samples for the mitochondria.

#### 4.1.4. HEp-2 Data Set

**Figure 6:**
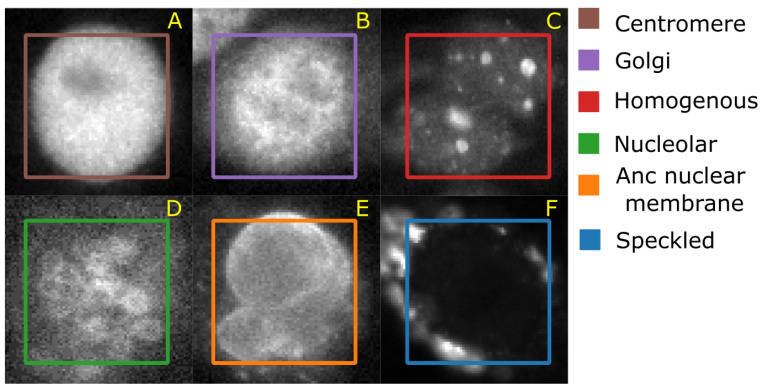
A representative image of each cell class and the average number of correctly classified instances in 10 cross validation. (**A**) Centromere (CT); (**B**) Golgi apparatus (GG); (**C**) Homogeneous (HM); (**D**) Nucleolar (NCL); (**E**) Anc nuclear membrane (ANM); (**F**) Speckled (SP).

The Human epithelial type-2 (HEp-2) cells were originally imaged by Sillivian Nicoliades pathology laboratory, Australia [42]. The data set was downloaded from Qi et al. [43] web-page where the authors also demonstrate an approach to classify the cells. The data set consists of 5 classes—centromere (CE), Golgi (GO), Homogeneous (HO), Nucleolar (NU), and nuclear membrane (ANC) and speckled (SP). Figure 6 shows representative images of each cell class. This data set contains 63,445 images of variable size annotated with ground truth labels, which are stored in a separate Matlab file. It should be noted that both HeLa and HEp-2 data sets are imbalanced.

### 4.2. Image Segmentation Task

The typical use of the segmentation functionality consists of 4 steps as follows: the first step is to provide defined samples of each class in different colors e.g., nucleous, background etc. as shown in Figure 2. The samples can be outlined using rectangular, polygon or composite regions of interest ROIs in ImageJ. Secondly, an array of geometric features at different scales are computed using the filters listed in Table 3 and a feature vector is generated for each pixel. In the third step, most discriminative features are selected using some useful heuristic—i.e., for example by Correlation-based Feature Selection (CFS) [44], which uses greedy hill climbing and best first search to explore the feature space. Various other feature selection techniques i.e., Principal Component Analysis (PCA), rank-based methods or others could also be used. The AS/IJ platform supports 2 feature selection algorithms—CFS and PCA. Next, the vectors are classified. For the purposes of the paper, in order to allow for comparisons with other platforms, the random forest classifier [45] with 100 default trees was used to train the model. The reasons behind using random forest as the default classifier for segmentation are: (1) it allowed us to compare our results with Ilastik [8]; (2) it has a smaller number of parameters and scales matching well with the size of the data set. Once the training is over, AS/IJ produces a class probability map, which is then thresholded and presented for user inspection. The corresponding mask is overlaid on the original image as shown in Figure 2.

For the purposes of comparison we have used the same filters as implemented in Ilastik. For the segmentation task, we have used two 2D topology-based segmentation metrics i.e., $V^{rand}$ and $V^{info}$ proposed by the challenge organizers themselves (see next section) [39]. $V^{rand}$ and $V^{info}$ are the normalized version of Rand error and information error after thinning the neurites border [46]. To compute the metrics we have performed 10 fold cross-validation using a random sample of 25 images for training and 5 for testing.

### 4.3. Segmentation Metrics

Evaluating results of segmentation is a non-trivial problem, which very much depends on the objective of the analysis. If the objective is correct segmentation of blobs then small errors of the boundary detection will not affect the desired result. In contrast, if pixel-wise error metric is used this situation may result in a large error bond. This has induced some authors to propose non-local or regional metrics. A systematic empirical study of this problem was performed in [7] where the authors recommended specially normalized versions of the “Rand error” $V^{rand}$ and “Variation of Information” $V^{info}$ which best matched expert judgments of segmentation quality for EM structure segmentation.

(10) $$V^{rand}:=2\frac{\sum_{i,j}p_{ij}^{2}}{\sum_{j}s_{j}^{2}+\sum_{j}t_{j}^{2}}$$

where $p_{i,j}$ is the probability that a randomly chosen pixel belongs to segment *i* of the boundary in *S* (segmented class) and segment *j* of the boundary in *T* (ground truth class). $s_{i}=\sum_{j}p_{i,j}$ is the marginal distribution density and denotes the probability of that a randomly chosen pixel belongs to segment *i* in *S*. $t_{j}=\sum_{i}p_{i,j}$ is the co-marginal distribution density and denotes the probability that a randomly chosen pixel belongs to segment *j* in *T*.

(11) $$V^{info}:=2\frac{I(S|T)}{H(S)+H(T)}$$

where

(12) $$I\left(S|T\right)=\sum_{i,j}p_{ij}\log p_{ij}-\sum_{i}s_{i}\log s_{i}-\sum_{j}t_{j}\log t_{j}$$

is the mutual information and H=−∑plogp is the corresponding entropy.

### 4.4. Image Classification Task

**Figure 7:**
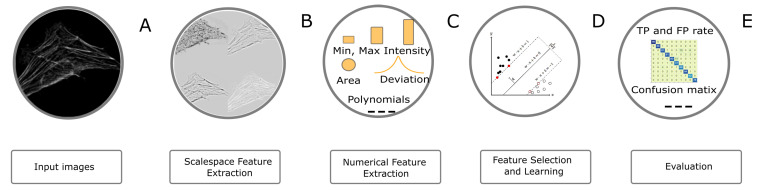
Overview of the Image Classification Workflow. (**A**) A sample image of filamentous actin labeled with rhodamine-phalloidin (Actin) from the HeLa data set; (**B**) Representative images of ALoG, LoG and BoG at scale 4; (**C**) Compound image transforms on original and scaled images, Feature vector is computed; (**D**) CFS based feature selection and SVM’s are used for training the model; (**E**) Several evaluation metrics are computed e.g., ROC curve, confusion matrix etc.

The image classification workflow is presented in Figure 7. In order to classify objects, we first enriched the feature space by extracting pixel features (i.e., LoG and ALoG) at several scales. The filters, features and parameters used for the task are summarized in Table 5 and Table 6. In a third step, we have extracted various regional features (Table 4). Instrumental for the practical applicability of this step is the feature selection, which was performed in the same way as in the segmentation workflow. Next, the SVM classifier was used to train the model. For the present task, we have used the SMO algorithm [34]. The outcome of the classification was evaluated against the ground truth by computing standard metrics—true positive rate (TP), false positive rate (FP), precision, recall, $F_{1}$ score, Receiver operating characteristic (ROC) area and Precision-Recall (PR) area.

For classification, we have used 10 fold cross validation and calculated several evaluation metrics in each iteration i.e., true positive (TP), false positive (FP), precision, recall, F1 score, area under ROC curve, area under PR curve. All classification metrics were computed pixel-wise (respectively voxel-wise).

## 5. Results

### 5.1. Image Segmentation

#### 5.1.1. Experiment 1: Batch Segmentation and Comparison

The first experiment used the ISBI training data set, for which expert-provided ground truth was present. For the purposes of comparison we have selected filters, which were implemented in Ilastik—LoG, Hessian of Gaussian, Structure Tensor—except for the ALoG filter. The scale space was computed for scales with filter half-widths ranging in 2,4,8 pixels. This was so, since the filters were computed in the spatial domain for odd window sizes. The objective of the experiment was to perform segmentation using pixel-level binary classification into cytoplasm and background (i.e., membranes and unclassified organelles). Table 7 shows training and testing results of the AS/IJ and Ilastik. Remarkably, both Ilastik and our platform achieved score 1.0 in $V^{rand}$ and $V^{info}$ metrics on the training data set. On the other hand, Ilastik outperformed AS/IJ by 0.04 in terms of $V^{rand}$, whereas $V^{info}$ was comparable for both platforms.

#### 5.1.2. Experiment 2: Three Class Segmentation

**Figure 8:**
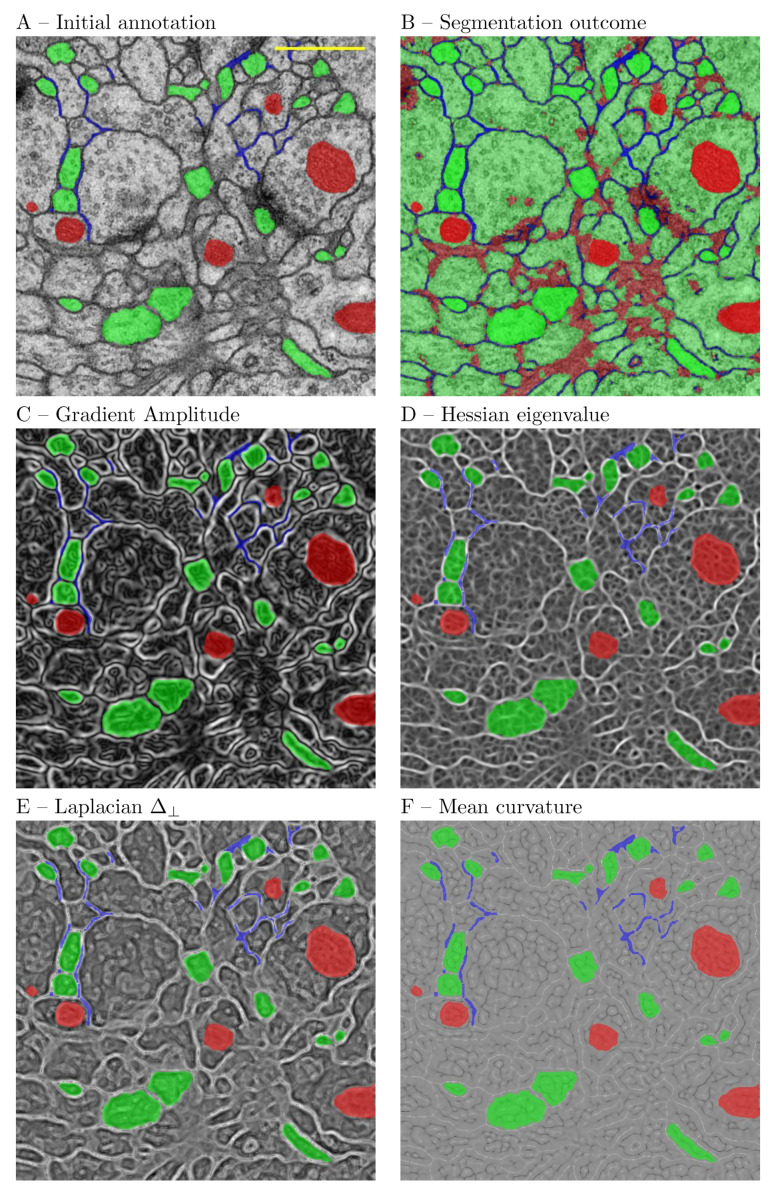
Characteristic features compared to the segmentation outcome. (**A**) Initial expert annotation. (**B**) Outcome of the segmentation; (**C**) Gradient amplitude, r=8; (**D**) Largest Hessian eigenvalue, r=8; (**E**) Normal component $\Delta_{⊥}$ of the LoG, r=8; (**F**) Mean curvature, r=8. The scale bar represents 0.5 μm.

**Figure 9:**
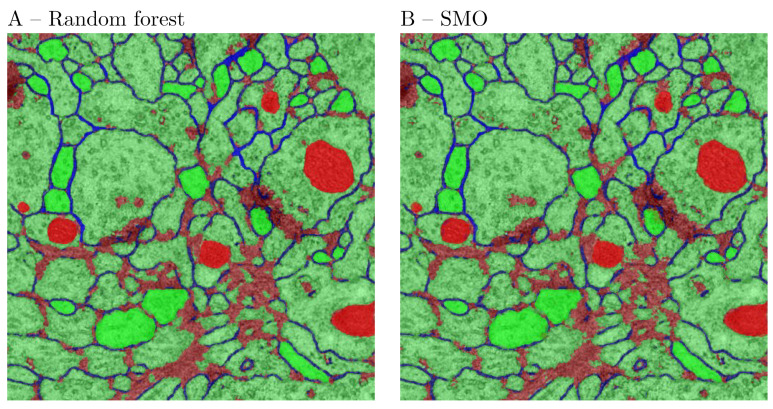
Characteristic features compared to the segmentation outcome. (**A**) RF classifier. (**B**) SMO classifier.

The second experiment used the ISBI testing data set, for which the ground truth is not publicly available. The first image of the data set was partially annotated for 3 types of structures—cell bodies, cell membranes and dark structures—representing presumably nuclei, vesicles and synaptic clefts. For this purpose we have used the 2D Gaussian, ALoG, Hessian, Structure Tensor, BoG and Curvature filters. The scale space was computed for the same range as above. The purpose of the annotation was to accurately outline the above structures to the extent possible for a neuroanatomist. The outcome of the experiment is presented in Figure 8. The initial feature set consisted of 82 features. The final features were selected using the PCA feature space reduction. The images were produced by the Feature Inspector functionality, which also exports the manual annotations. The most prominent features from the perspective of the human visual system were discerned at the largest scale r=8. Cell membranes could be correlated with the normal component of the Laplacian $\Delta_{⊥}$, the Hessian eigenvalue $E_{1}$, and the mean curvature. The nucleoplasm could be correlated with features on smaller scales. In a different trial the SMO classifier was compared to the random forest (RF). The RF classifier learned correctly 100% of the presented pixels (29,468) with root mean squared error (RMS) of 0.0616. The SMO classifier learned correctly 91.56% of the presented pixels (29,468) with RMS error of 0.309. The results are presented in Figure 9 and Table 8. From the figure it is apparent that both classifiers resulted in comparable segmentation outcomes. Therefore, it could be concluded that the RF classifier over-fitted the data. On the other hand, the classification by random forest took considerably more time. Therefore, for such a task the SMO classifier is recommended.

### 5.2. Image Classification

#### 5.2.1. Case Study 1: Synthetic Data Set Classification

On the first place, using the synthetic image set AS/IJ was trained with 100 examples of circles and triangles and test it on 50 samples of each. We were able to achieve 100% accuracy $R_{1}$ score, area under ROC curve and area under PR curve, using the SVM classifier, for both the test and training data sets. This was to be expected as the data set emphasized the differences in the properties of the Zernike moments compared to the Legendre moments.

#### 5.2.2. Case Study 2: HeLa Cells Classification

**Figure 10:**
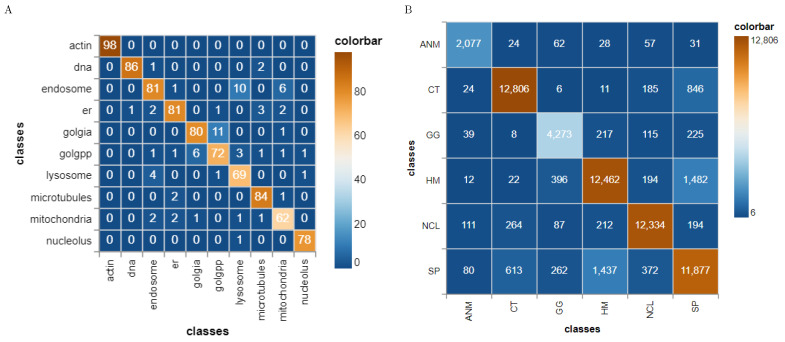
Confusion matrix of 10 fold cross validation for HeLa (**A**) and HEp2 (**B**) using SVM, regional and scale space features and feature selection. (**A**) HeLa data set, class annotations are the same as in Figure 5; (**B**) HEp2 data set, class annotations are the same as in Figure 6.

Classification results were analyzed by computing the confusion matrix. Figure 10 demonstrates the high level of confusion between the Golgi protein giantin (Golgi) and Golgi protein GPP130 (Golgpp). Note that these protein structures also look similar when inspecting the raw images. The $F_{1}$ scores of our method with feature selection is 3% higher than the results reported by the wnd-charm method [14]. Our approach also achieved 90.2% area under PR curve and 98.8% area under ROC curve, respectively.

#### 5.2.3. Case Study 3: HEp2 Cells Classification

Presented classification workflow Section 4.4 worked well even with a large data set, such as the HEp2 images. $F_{1}$ scores of our method with feature selection is 14% higher than the results reported by wnd-charm method and very much near to the neural network method [14]. We have also achieved 83.0% area under PR curve and 95.0% area under ROC curve, respectively. Table 9 shows the comparison among three different classification scenarios. The first scenario used regional features only, consisting 203 features in total. The second scenario used a combination of regional features and scale space features, consisting of 5887 features. The third scenario used feature selection during learning. The main problem encountered during incorporating scale space features is that the feature space increases exponentially for a large data set, such as HEp-2. This was the reason while HEp-2 classification could not be computed in the second scenario. The feature selection step used in the third scenario helped to overcome this problem. In total, only 142 and 173 features were selected for HeLa and HEp-2 data sets using the CFS method, respectively. Interestingly, features are selected from both the higher and lower scales. In this regard, we could comment only on the anisotropic Lagrangian ALoG because of its clear geometrical meaning. In case of ALoG, tangential components are selected for some small scales which corresponds to fiber-like structures, while orthogonal components are selected from the larger scale, which corresponds to blob-like structures.

### 5.3. Influence of the Classification Method

In a different experiment, we compared the SVM vs. Random Forest (RF) classifiers on the HeLa data set. We used only the baseline of regional features and the ImageJ statistics (Table 4). The RF classifier was run with 100 iterations and base learner settings. The results are presented in Table 10. From the data it is apparent that the SVM classifier performed better than the RF classifier on the selected data set.

## 6. Discussion

The approach presented in this article integrates geometrical feature extraction based on signal processing and machine learning with human expert input relying on domain knowledge. The main advantage of AS/IJ is the pluggable filter functionality, which is designed in the same spirit as ImageJ. Another advantage of AS/IJ is the rich family of filters that are distributed with the platform. Specifically, the Gaussian Jet, LoGN, ALoG and Curvature filters are not present in other packages to date.

### 6.1. Platform Comparison

The AS/IJ platform is of the same class as two other platforms – TWS and Ilastik. However, there are notable differences in the image processing functionality. Ilastik implements the following filters: ST eigenvalues, Hessian eigenvalues, LoG, Amplitude of Gradient, Difference of Gaussians (DoG) and Gaussian.

Ilastik also supports extendability by plugins, however the filter set at present is still limited. Compared to Ilastik, AS/IJ is distributed with more filters implementing scale space theory—notably Bi-Laplacian, Gaussian Jet, Curvatures in 2D (line) and 3D (surface) and ALoG. The first order gradient filter is complete in the sense that it produces both coordinate components as well as the amplitude/phase decomposition, while in Ilastik only the amplitude is computed.

TWS features a more varied filter collection, which have been selected mostly for historical reasons. These are notably the Gaussian, Hessian, Membrane projections, Ranking filers (mean, max, min, median), variance, entropy, Anisotropic Diffusion, Lipschitz, Gabor, LoG, Sobel, DoG, Kuwahara, Gaussian Derivatives (up to order 5), ST. The TWS platform limits the choice of filters only to hard-coded ones. The Sobel filter implements a gradient operation. Both the Kuwahara and Anisotropic Diffusion filters are edge-preserving smoothing filters, which are more suitable for image preprocessing. The Lipschitz implements the Lipshitz cover, which is a morphological operation, suitable for eliminating slowly-varying image background. As such this filter could be more useful for enhancing natural images or bright microscopic images, where the illumination field is uneven. The local statistic filters (i.e variance and entropy) output information about the local feature variation. As a result the range of applications of TWS is also more limited than Ilastik and AS/IJ. Moreover, the platform is geared mostly towards segmentation.

The DoG functionality, present in both TWS and Ilastik is completely superfluous, as it was introduced as an approximation of LoG in the times when computational power and memory for personal computing were very limited. This is the reason why it is not present in AS/IJ. From this perspective, it can be used mostly for historical reasons for comparing results with legacy code.

AS/IJ is therefore, the platform which implements more completely the functionality prescribed by differential geometry. Multiscale morphological image filers (e.g., ranking, opening, closing) were not included in the present release of the platform, although such filters are present in ImageJ, for the following reason. The filters are not separable and, therefore, computational time scales as $O(N^{k})$ for a *k*-dimensional image. For large images the performance penalty could become appreciable.

A major difference with regard to the TWS is the interactive feature inspection functionality. In our experience this can be useful in two scenarios. On the frist place, when refining ROIs, one can reshape them to cover filter responses that are more discriminative for the problem at hand. This is facilitated by the functionality to overlay the ROI annotations on the filter response (Figure 8). On the second place, based on an initial expert choice of filters and the outcome of the initial segmentation, one can decide either to exclude some or include other filters and retrain the system. The metadata concept is another major difference with IWS and Ilastik, which at present do not support metadata.

### 6.2. The Problem of Ground Truth

The most common bottleneck in most of the segmentation problems is labeling of ground truth for training. In most cases, the ground truth must be provided by experts (i.e., trained neuroanatomists or pathologists). This is a substantial impediment for big-data approaches as ground truth annotation becomes very time-consuming and expensive. Another problem is that for many images it is not possible to delineate with certainty the structures of interest. Therefore, in many cases one has to deal with only partial ground truth. In our view, this is the norm and not the exception. Therefore, any segmentation workflow should explicitly address this issue. Although implicit, such is the understanding when using TWS, which for its time became a landmark innovation in the interactive segmentation [7]. Newer tools, such as Ilastik, also embrace this assumption. In our view, it does not look reasonable that deep learning approaches would immediately alleviate the above situation. One could envision an approach where AS/IJ is used to augment the ground truth provided by experts and use it for training of deep learning CNNs.

### 6.3. The Problem of Feature Selection

Machine learning offers a systematic approach, where predefined features are combined into different classifiers. On the other hand, the outcome of machine learning is only as good as the underlying feature space. This can draw some criticism from the proponents of an alternative approach where the features are learned from the image set. The criticism has a point in the sense that simply adding more “hand-crafted” features may not be very useful. On the other hand, one can readily address such criticism by resorting to some generic mathematical theory. The present work supports the view that differential geometry can substantially improve the outcome of machine learning since it can enrich the underlying feature space with new invariant objects. We have used differential geometry to enrich the feature space by adding more integration scales but also have constrained the problem by using only invariant features, which do not depend on the representation. Moreover, these features are linearly independent and, therefore, not trivial to learn from data. The main methodological innovation of the present approach is the use of the orthogonal decomposition of the Laplacian, implemented as the ALoG filter, Equations (A2) and (A3), which allows for treatment of anisotropic data and to capture directional information.

In our experience, for blob-like structures, such as nuclei or cytoplasmic vesicles, ALoG alone only gives acceptable segmentation results. For more challenging data sets with varying textures one can use the combinations of ALoG and Hessian or even the full set of filters.

### 6.4. Image Classification Aspects

Presented results demonstrate the benefits of using geometric features for image classification in the framework of scale space theory. It is demonstrated that scale and feature selection approaches can overcome the exponential increase of training time that otherwise can be entailed by simply enriching the feature space (Table 9). Most of the instance classification methods in literature do not have this feature enrichment step, as they directly extract texture features [14]. It should be noted that the SVM and RF classifiers are used here merely for comparison purposes. One can also use any other classifier available in the Weka library.

### 6.5. Outlook

The present paper discusses only four limited use cases where comparison with other platforms helps situate the presented work. However, this by no means exhausts the possible applications of AS/IJ. An interesting future direction of research using the platform will be to explore feature spaces and compare them to intermediate outcomes of convolution neural networks (CNNs) where image-driven features are computed. This could potentially offer more insights into the operation of CNNs. This can be achieved by comparisons with platforms, such as Cellpose [47] and StarDist [48]. Another interesting direction of research, which is currently in progress, is to investigate α-scale spaces and to also make available differential invariants based on α-scale space theory [30].

### 6.6. Conclusions

The main utility of the presented multi-scale approaches is to build a multidimensional feature space, which is subsequently used to learn characteristic “fingerprints” of the objects of interest. In summary, the results obtained in the image classification tasks indicate that feature space enrichment properly balanced with feature reduction can achieve performance comparable to deep learning approaches. Therefore, differential geometry can substantially improve the outcome of machine learning since it can enrich the underlying feature space with new geometrical invariant objects. In conclusion, the present paper demonstrated a new platform for image segmentation and classification supporting an extendable filter set. The platform has a very broad application range, as demonstrated by the presented use cases, and can become a valuable contribution to the ImageJ ecosystem of tools.

## Figures and Tables

**Table 1:** Low-order differential invariants.

First Order Invariants	
Gradient amplitude	$A=\sqrt{G_{x}^{2}+G_{y}^{2}}$
Gradient orientation	$\sin\phi =G_{y}/\sqrt{G_{x}^{2}+G_{y}^{2}}$
	$\cos\phi =G_{x}/\sqrt{G_{x}^{2}+G_{y}^{2}}$
**Second order invariants**	
Laplacian	$\Delta_{G}=\mathrm{Tr}\;H_{G}=G_{xx}+G_{yy}$
Determinant of the Hessian	$\det\;H_{G}=G_{xx}G_{yy}-G_{xy}^{2}$

**Table 2:** Curvature invariants.

Curvature Invariant	Formula
Line curvature	$\left(G_{xx}G_{y}^{2}-2G_{x}G_{y}G_{xy}+G_{x}^{2}G_{yy}\right)/{\left(G_{x}^{2}+G_{y}^{2}\right)}^{3/2}$
Mean curvature	$1/2\left(\left(1+G_{x}^{2}\right)G_{yy}-2G_{x}G_{y}G_{xy}+\left(1+G_{y}^{2}\right)G_{xx}\right)/{\left(1+G_{x}^{2}+G_{y}^{2}\right)}^{3/2}$
Gaussian curvature	$\left(G_{xx}G_{yy}-G_{xy}^{2}\right)/{\left(1+G_{x}^{2}+G_{y}^{2}\right)}^{2}$

**Table 3:** Filters computing geometric features.

Filter	Functionality	Feature Order
Gauss2D	Gaussian smoothing, Equation (4)	0
Gradient	Gradient amplitude and orientation	1
Gaussian Structure	Structure tensor, Equation (2)	1
LoG	Laplacian of Gaussian (LoG)	2
ALoG	Anisotropic decomposition of LoG, Equations (A2) and (A3)	2
	Gradient amplitude and orientation	1
Hessian	Eigenvalues of the Hessian	2
	Determinant of the Hessian	2
Curvature 2D	Line curvature + Hessian determinant	2
Curvature 3D	Mean + Gauss curvature of surfaces	2
BoG	Bi-Laplacian of Gaussian, Equation (5)	4
Gaussian Jet	Gaussian Jet of order *n*	n
LoGN	*n*-th order PoL, Equation (5)	2 n

**Table 4:** ImageJ plugins used for computation of texture features.

Regional Feature	Functionality
Legendre	Legendre moments
Zernike	Zernike moments
ImageJ	ImageJ statistics
Haralick	Haralick features [12]

**Table 5:** Scale space filters and scales used for Hela and HEp-2 data sets.

Data Set	Filters	Min Scale	Max Scale
HeLa	LoG, ALoG, HoG, Structure	2	8
HEp-2	LoG, BoG, ALoG, HoG, Structure	2	8

**Table 6:** Regional Features and parameters used for Hela and HEp-2 data sets.

Regional Feature	Parameters
Legendre	order (0–6)
Zernike	order (0–6)
ImageJ	–
Haralick	distance (1–3), directions (90${}^{\circ }$, 180${}^{\circ }$, 270${}^{\circ }$, 360${}^{\circ }$)

**Table 7:** Comparative performance of AS/IJ and Ilastik on the ISBI 2012 data set.

Metric	Training Set		Test Set	
	AS/IJ	Ilastik	AS/IJ	Ilastik
$V^{rand}$	1.0	1.0	0.87	0.91
$V^{info}$	1.0	1.0	0.93	0.94

**Table 8:** SMO learning performance in 3 class segmentation task.

TP Rate	FP Rate	Precision	Recall	$F_{1}$	ROC Area	PRC Area	Class
0.954	0.081	0.942	0.954	0.948	0.943	0.932	cells
0.722	0.020	0.808	0.722	0.763	0.935	0.667	boundaries
0.909	0.048	0.897	0.909	0.903	0.934	0.847	nuclei

**Table 9:** Comparison of mean average true positive rate (TP), false positive rate (FP), precision, recall, $F_{1}$ score, ROC area and PR area of different classification approaches in different data sets.

Data Set	TP	FP	Precision	Recall	$F_{1}$	ROC Area	PRC Area
Regional Features							
Hela	0.82	0.02	0.82	0.81	0.81	0.96	0.75
HEp-2	0.76	0.06	0.77	0.76	0.76	0.90	0.68
Regional features + Scale space pixel-features							
Hela	0.92	0.01	0.92	0.92	0.92	0.98	0.88
HEp-2	–	–	–	–	–	–	–
Regional features + Scale space pixel-features+ discriminative features selection							
Hela	0.93	0.01	0.93	0.93	0.93	0.99	0.90
HEp-2	0.88	0.03	0.88	0.88	0.88	0.95	0.83

**Table 10:** Comparison of SVM and Random Forest (RF) classifiers on the HeLa data set.

Classifier	TP	FP	Precision	Recall	$F_{1}$	ROC Area	PRC Area
SMO	0.806	0.021	0.805	0.806	0.804	0.955	0.729
RF	0.717	0.031	0.712	0.717	0.713	0.950	0.758

## Data Availability

A stable release of Active Segmentation can be downloaded from https://zenodo.org/record/5770592. The data sets analysed in the present paper were downloaded from the following open source repositories: HeLa data set https://murphylab.web.cmu.edu/data/2Dhela_images.html (accessed on 1 February 2021), Hep2 data set http://qixianbiao.github.io/HEp2Cell/ (accessed on 1 February 2021), and ISBI 2012 EM data set https://imagej.net/events/isbi-2012-segmentation-challenge (accessed on 28 April 2018). The data set, demonstrating Figure 8, can be downloaded from https://zenodo.org/record/5771719.

## Appendix A. Weak Differentiation

Differentiation in the literal sense is not applicable to discrete signals. This difficulty can be overcome by applying the distribution theory [19], starting from the Leibniz identity for smooth signals *I* and *G*

$$\nabla \left(I*G\right)=\left(\nabla I\right)*G+I*\nabla G,\;I,G\subset E^{2}$$

where *∇* represents the gradient given by its principal components ∇=(∂/∂x,∂/∂y). For the whole space if the kernel vanishes sufficiently fast at infinity it follows that

(∇I)∗G=−I∗∇G

The weak Hessian tensor with respect to the kernel G is defined as

$$H_{G}\left(I\right):=\left(\begin{array}{cc} G_{xx} & G_{xy}\\ G_{xy} & G_{yy} \end{array}\right)*I$$

## Appendix B. Orthogonal Laplacian Decomposition

The Laplace operator can be decomposed in two orthogonal components—on in the direction of the isophote and the other in the direction normal to the isophotes. The representation is provided below for reference:

(A1) $$\begin{array}{cc} \Delta_{G} & =\Delta_{||G}+\Delta_{⊥G}\\ \left(G_{x}^{2}+G_{y}^{2}\right)\Delta_{⊥G} & =\left(G_{x}^{2}\right)G_{xx}+\left(2G_{x}G_{y}\right)G_{xy}+\left(G_{y}^{2}\right)G_{yy}\\ \left(G_{x}^{2}+G_{y}^{2}\right)\Delta_{||G} & =\left(G_{x}^{2}\right)G_{xx}-\left(2G_{x}G_{y}\right)G_{xy}+\left(G_{y}^{2}\right)G_{yy} \end{array}$$

Here, *G* is a shorthand of a convolution with a Gaussian kernel or its derivative of an appropriate order. This is implemented as the ALoG filter (see Table 3).
